# Arsenic Trioxide Induces Apoptosis via Specific Signaling Pathways in HT-29 Colon Cancer Cells

**Published:** 2016-01-09

**Authors:** Jacqueline J Stevens, Barbara Graham, Erika Dugo, Bezawit Berhaneselassie-Sumner, Kenneth Ndebele, Paul B Tchounwou

**Affiliations:** 1 Molecular and Cellular Biology Research Laboratory, NIH RCMI-Center for Environmental Health, College of Science, Engineering and Technology, Jackson State University, Jackson, MS, USA; 2 Molecular Toxicology Research Laboratory, NIH RCMI-Center for Environmental Health, College of Science, Engineering and Technology, Jackson State University, Jackson, MS, USA

**Keywords:** Arsenic trioxide, Apoptosis, Oxidative stress, Mitochondria apoptotic pathway, HT-29 human colorectal adenocarcinoma cells

## Abstract

**Background:**

Arsenic trioxide (ATO) is highly effective in the treatment of patients with acute promyelocytic leukemia (APL). It is a chemotherapeutic agent that has been shown to induce apoptosis in several tumor cell lines. However, research into its effects on colon carcinoma cells is still very limited. We previously reported that ATO is cytotoxic and causes DNA damage in HT-29 human colorectal adenocarcinoma cells. In the present study, we further evaluated its effect on oxidative stress (OS), and examined its apoptotic mechanisms of action on HT-29 cells.

**Methods:**

OS was assessed by spectrophotometric measurements of MDA levels while cell cycle analysis was evaluated by flow cytometry to determine whether ATO induces cell cycle arrest. Its effect on early apoptosis was also evaluated by flow cytometry using Annexin V-FITC/PI staining. Fluorescence microscopy was used to detect the morphological changes, and Western blotting was carried out to determine the expression of apoptosis-related proteins.

**Results:**

The lipid peroxidation assay revealed a dose-dependent increase in MDA production. DAPI staining showed morphological changes in the cell’s nucleus due to apoptosis. Cell cycle analysis and Annexin V-FITC assay also demonstrated a dose-dependent effect of ATO in the accumulation of cells at the sub G1 phase, and the percentages of Annexin V-positive cells, respectively. Western blot data showed that ATO upregulated the expression of caspase 3, Bax, and cytochrome C, and down-regulated the expression of Bcl-2.

**Conclusion:**

Taken together, our findings indicate that ATO induces OS and cytotoxicity in HT-29 cells through the mitochondria mediated intrinsic pathway of apoptosis.

## Introduction

Colorectal cancer (CRC) is the third leading cause of cancer-related deaths in the United States. It is the most frequent cancer in men, after lung and prostate cancer, and is the second most frequent cancer in women after breast cancer [1–3]. CRC represents approximately 10% of cancer-related deaths [2,3]. Multiple risk factors including smoking, alcohol consumption, dietary factors, family history, lifestyle, ethnicity, and genetic alterations have been associated with colon cancer [1–4]. The outcome of colon cancer has improved with early diagnosis and surgical intervention combined with other treatments such as chemotherapy, radiotherapy or targeted therapy [5,6]. Radiotherapy and chemotherapy using cytotoxic drugs are the major methods of cancer treatment, and many anticancer drugs have been applied clinically for colon cancer [5,6]. Chemotherapy is a common treatment option for patients with stage III or stage IV colorectal cancer. However, there are very few effective strategies available to treat metastatic colon cancer or tumor recurrence. Current chemotherapeutic regimens for CRC are represented by fluoropyrimidine-based treatments such as 5-fluorouracil (5FU), cetuximab, panitumumab, paclitaxel, docetaxel, vincristine and oxaliplatin [7–9]. Due to the increased concerns related to side effects and drug resistance associated with these current treatments, more effective drugs to treat colorectal carcinoma are necessary as potential anticancer agents. Nevertheless, the side effects of these therapies are severe. Therefore, it is important to identify potential chemotherapeutic agents with stronger antitumor effects and investigate their mechanisms of antitumor activity.

Arsenic compounds have been active components in both Western and traditional Chinese medicines that can be dated back more than two thousand years ago [10]. The medicinal effect of ATO is of great significance, though, it is also well known for its toxicity. Arsenic trioxide has antitumor activities in several cancers and the antitumor activities have been correlated with its ability to inhibit cell proliferation and induce apoptosis [10–14]. Recently, it has become one of the most effective anticancer drugs, especially in the treatment of acute promyelocytic leukemia [14–16]. The therapeutic potential of ATO is not just limited to treating APL, but this drug has been used in the treatment of several human diseases including malaria, psoriasis, syphilis, rheumatosis and cancer [10–12]. Numerous studies have documented the use of ATO in the treatment of various malignancies [14–18] including B-cell lymphoma and multiple myelomas clinical trials [18–22]. Previous *in vitro* studies have indicated that ATO may be effective on solid tumors such as in human pancreatic (AsPC-1), colonic (HT-29), lung (A549), breast (MCF-7), neuroblastoma, head and neck cancer cells, gastrointestinal and liver carcinoma (HepG2) cells [11,23–30].

Apoptosis (programmed cell death) is a normal developmental process that results in cell death. It is characterized by nuclear condensation and cleavage of critical cellular proteins. Apoptosis has been known to play a role in maintaining normal development and homeostasis in multicellular organisms and allowing organisms to respond appropriately to environmental stimuli [13]. Apoptosis can be triggered through an extrinsic (death receptors) or intrinsic (mitochondrial) pathway. In the intrinsic pathway, mitochondria act as central integrators of apoptosis and are characterized by disruption of mitochondrial membrane potential, release of pro-apoptotic proteins into the cytosol (e.g. Cyt c, BID, Bax), subsequent caspase cascade activation, DNA fragmentation, chromatin condensation, and cell shrinkage [31]. Hence, we focus on the apoptotic mechanisms triggered by ATO in colon-cancer cells. The medicinal effect of ATO is of great significance, though, it is also well known for its toxicity. Arsenic trioxide has been shown to exert its therapeutic effect through different cellular and physiological pathways; however the associated mechanisms of action are not clearly understood. Preclinical studies have demonstrated that ATO can induce apoptosis and inhibit cell growth in a wide variety of tumors [28–30]. Published research has reported that ATO influences multiple pathways, which may result in the induction of apoptosis, genotoxicity, enhanced cell proliferation, promotion of differentiation, oxidative stress and activation or inhibition of a variety of cellular signal transduction pathways [13,32]. Through the process of apoptosis, ATO normally eliminates damaged or unwanted cells from organisms, which causes the cellular dysfunction in malignant cells, thus resulting in more benefits for future cancer therapy [13]. Arsenic trioxide induces apoptosis mainly through activating the mitochondria-mediated intrinsic apoptotic pathway [16,33]. Arsenic trioxide affects the activities of caspases and pro- and anti-apoptotic proteins. The down-regulation of Bcl-2, an “anti-apoptotic” protein, has been considered as one of the significant mechanisms of action [34]. The Bcl-2 family of proteins is comprised of proapoptotic and anti-apoptotic proteins that play a pivotal role in the regulation of apoptosis, especially via the intrinsic pathway as they reside upstream of irreversible cellular damage and act mainly at the mitochondria level [35]. The Bcl-2 family proteins are key regulators of apoptosis cell death. Studies have shown that ATO initiated apoptosis by activating the mitochondria apoptotic pathway as indicated by the inhibited Bcl-2 expression, release of cytochrome c and activation of caspase cascade (e.g. caspase 3, caspase 9 and Bax) in several cell lines [35–40]. Recent studies conducted in our laboratory have demonstrated that ATO is cytotoxic and genotoxic as revealed by the significant increase in DNA damage in HT-29 cells [41,42]. The present study was designed to further investigate its effects on OS and to examine its apoptotic mechanisms of action on HT-29 cells.

## Materials and Methods

### Cell culture and treatments

The HT-29 human colorectal adenocarcinoma cells were obtained from the American type culture collection (ATCC) (Manassas, VA). Cells were maintained in a CO_2_ incubator at 37°C under a humidified atmosphere (95% air, 5% CO_2_) in McCoy’s 5A growth medium supplemented with 10% FBS and 1% antibiotics (penicillin/streptomycin). Cells were treated with several concentrations of ATO (0, 2, 4, 6, 8 and 10 μg/ml) for 24 h to elucidate the underlying mechanisms of apoptosis-induced cell death. Only sub-confluent monolayers of cells (70% to 80%) were used in all experiments.

### Chemicals and reagents

McCoy’s 5A growth medium, phosphate buffered saline (PBS), and trypan blue were purchased from ATCC (Manassas, VA). Fetal bovine serum (FBS), penicillin/streptomycin, and 0.25% trypsin-EDTA (w/v) were purchased from Gibco (Grand Island, NY). Arsenic trioxide (lot # 02765-24) and Nunc TM Lab-TekTM chamber slides were purchased from Fisher Scientific (Houston, TX). A lipid peroxidation kit was purchased from Abcam (Cambridge, MA). Prolong Gold Antifade Reagent with 4′,6-diamidine phenylindone (DAPI) solution was purchased from Invitrogen Corporation (Grand Island, NY). ECL western blotting detection system reagents and Films (CL-X-posure) were purchased from Thermo Scientific/Pierce (Rockford, IL). Pre-cast gels were purchased from Fisher Scientific (Pittsburg, PA). The folin-phenol (DC) protein determination kit, cell lysis buffer, and non-fat milk were obtained from BioRad Laboratories (Hercules, CA) and, polyvinylidene difluoride (PVDF) membrane was obtained from Millipore (Bedford, MA). The primary antibodies anti-caspase 3 and anti-**cytochrome c** were purchased from Cell Signal Technology, Inc (Danvers, MA). The primary antibodies anti-Bcl-2, anti-Bax, and horseradish peroxidase (HRP) conjugated anti-mouse or anti-rabbit goat secondary antibodies and β-actin were purchased from EMD Millipore Corporation (Billerica, MA).

### Lipid peroxidation assay

HT-29 cells were treated with or without ATO and lipid peroxidation was evaluated by measuring malondialdehyde (MDA) levels using the lipid peroxidation assay kit (Abcam) as previously described [43]. The amount of MDA formed in each of the samples was assessed by measuring the absorbance of the supernatant at 586 nm with a UNICCO 2800 UV/VIS Spectrophotometer (Thermo Fisher, Waltham, MA).

### Cell cycle analysis by flow cytometry

Phase distribution of the cell cycle was determined by flow cytometry. Cell cycle analysis was performed as described by Ndebele et al. [44]. HT-29 cells were cultured in 13 × 100 mm plates at a density of 3 × 10^5^/plate. The cells were treated with ATO at various concentrations for 24 h. Control cells (untreated) were cultured in medium alone. The cells were washed twice with cold PBS and harvested. The cell pellets were suspended in 250 μL of propidium iodide (PI) solution and incubated at 4°C in the dark for 1 h at a density of 1 × 10^6^ cells/ml. The cell cycle distribution was measured using FACS Vantage flow cytometry system and Cell-Quest software (Becton-Dickinson, San Jose, CA).

### Nuclear staining with 4′,6-diamidino phenylindole (DAPI)

4′,6-diamidino phenylindole (DAPI) staining was used to observe the morphology changes of the nuclei of HT-29 cells treated with various concentrations of ATO. Cells were seeded at a density of 3 × 10^4^ cells/ml and cultured in one-chamber glass slides (Lab-Tek, Nunc, Naperville, IL). After ATO treatment for 24 h, the slides were then rinsed with PBS and fixed in PBS containing 3.7% paraformaldehyde for 30 min at room temperature, washed twice with PBS and stained with Prolong Gold anti-fade with DAPI (1 μg/ml) and placed in the dark for 24 h. The stained nuclei were observed under a fluorescence microscope (Olympus) with the appropriate filter.

### Analysis of apoptosis using Annexin V sand PI staining

The Annexin V-FITC/PI assay was conducted to further assess the effect of ATO on cell death through apoptosis [45,46]. HT-29 cells were cultured in 13 × 100 mm plates at a density of 3 × 10^5^. The cells were treated with ATO at various concentrations for 24 h and collected by trypsinization. Untreated cells served as negative controls. The cells were washed with PBS and diluted in 1X Annexin binding buffer (100 μL). For each sample, 5 μL (2.5 μg/ml) of Annexin-V-FITC and 5 μL (50 μg/ml) propidium iodide (Roche Scientific, Indianapolis, IN) were added to cell suspension and incubated for 15 min at room temperature (25°C) in the dark. An additional 400 μL of Annexin binding buffer was added to each sample for a total of 500 μL. Cell death by apoptosis was scored by quantifying the population of Annexin V-FITC-positive cells (10,000 events). Flow cytometry data were plotted and analyzed by the fluorescence activated cell-sorting (FACS-Vantage) system using the Cell quest software (Becton-Dickinson, San Jose, CA) within 1 h of staining.

### Western Blot analysis

Protein expression of Bcl-2, Bax, cytochrome C and caspase 3 was determined using Western blotting as previously described [47]. HT-29 cells were cultured in 13 × 100 mm plates at a density of 3 × 10^5^. The cells were treated with ATO at various concentrations for 24 h, collected by trypsinization and lysed in lysis buffer [20 mM Tris-HCl (pH 7.5), 150 mM NaCl, 1 mM Na_2_EDTA, 1 mM EGTA, 1% Triton, 2.5 mM sodium pyrophosphate, 1 mM B-glycerophosphate, 1 mM Na_3_VO_4_ and 1 μg/ml leupeptin in the presence of a protease inhibitor (Thermo Scientific, Rockford, IL). Protein concentrations were determined using the Bio Rad Dc Protein Assay Kit (Hercules, CA). Subsequently, equivalent amounts of proteins were separated by sodium dodecyl sulfate-polyacrylamide gel electrophoresis (SDS-PAGE), transferred to polyvinylidene fluoride (PVDF) membranes (Bio-Rad Laboratories, Hercules, USA) using the Trans-Blot^®^ Turbo^™^ Transfer System (Bio-Rad) and then blocked in 10% non-fat milk (Bio-Rad) containing 0.05% Tween-20 (PBST) and incubated at room temperature for 1 h. The membranes were probed with various primary antibodies against caspase-3 (Cell Signaling, Danvers, MA), Bcl-2 (EMD Millipore Corporation, Billerica, MA), Bax (EMD Millipore Corporation, Billerica, MA) and cytochrome C (Cell Signaling, Danvers, MA) and β-actin (1:1,000) (Cell Signaling, Danvers, MA) at 4°C overnight. After the PVDF membranes were washed three times in PBST, incubated with horse radish peroxidase (HRP)-conjugated secondary antibodies (1:10,000) at room temperature for 1 h and the membranes were washed again in PBST three times. Immunoreactive proteins were detected with enhanced chemiluminescence (ECL-plus) western blotting detection system (Thermo-Fisher Scientific) followed by exposure to CL-Xposure film (Thermo-Fisher Scientific/Pierce) and visualized according to manufacturer’s instructions. Densitometry analysis of X-ray films was performed using the Molecular Imager (Bio Rad) (Quantity One, Version 4.67 software). To assess the presence of comparable amount of proteins in each lane, β-actin was used as a loading control.

### Statistical analysis

Statistical analyses of data were performed using a student’s t-test and ANOVA to evaluate differences between test samples and controls. Each experimental condition was performed in triplicate (n=3), and all data were expressed as means ± standard deviations (SD). All p-values of <0.05 were considered to be statistically significant. Oxidative stress and apoptotic data were presented graphically in the form of histograms, using Microsoft Excel computer program to represent the dose-response relationship among the treatment groups.

## Results

### Effect of ATO on oxidative stress in HT-29 Cells

**Figure 1:**
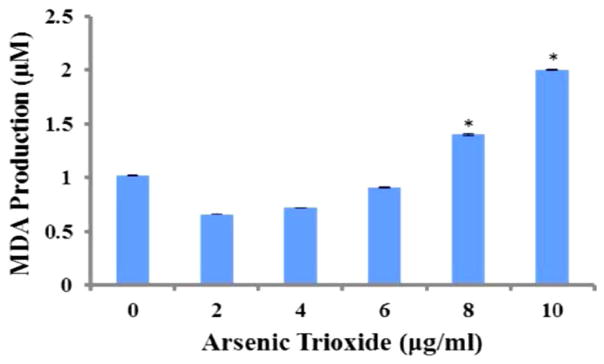
Effect of ATO on oxidative stress in HT-29 cells. The MDA levels were 1.02 ± 0.005, 0.66 ± 0.001, 0.72 ± 0.001, 0.91 ± 0.007, 1.4 ± 0.006, and 2.0 ± 0.006 μM for 0, 2, 4, 6, 8 and 10 μg/ml, respectively. *The data revealed significant differences (p<0.05) in MDA production at 8 μg/ml and 10 μg/ml when compared to the control (0 μg/ml).

Lipid peroxidation assay was used to assess OS by measuring the amount of malondialdehyde (MDA) in controls and ATO-treated HT 29 cells. The test results indicated a dose-dependent increase in MDA production. The MDA concentrations were 1.02 μM ± 0.005 μM, 0.66 μM ± 0.001 μM, 0.72 μM ± 0.001 μM, 0.91 μM ± 0.007 μM, 1.4 μM ± 0.006 μM, and 2.0 μM ± 0.006 μM for 0, 2, 4, 6, 8 and 10 μg/ml, respectively. As shown in Figure 1, the data revealed significant differences (p<0.05) in MDA production at 8 μg/ml and 10 μg/ml compared to the control (0 μg/ml).

### Effects of ATO on cell cycle and apoptosis in HT-29 cells

**Figure 2:**
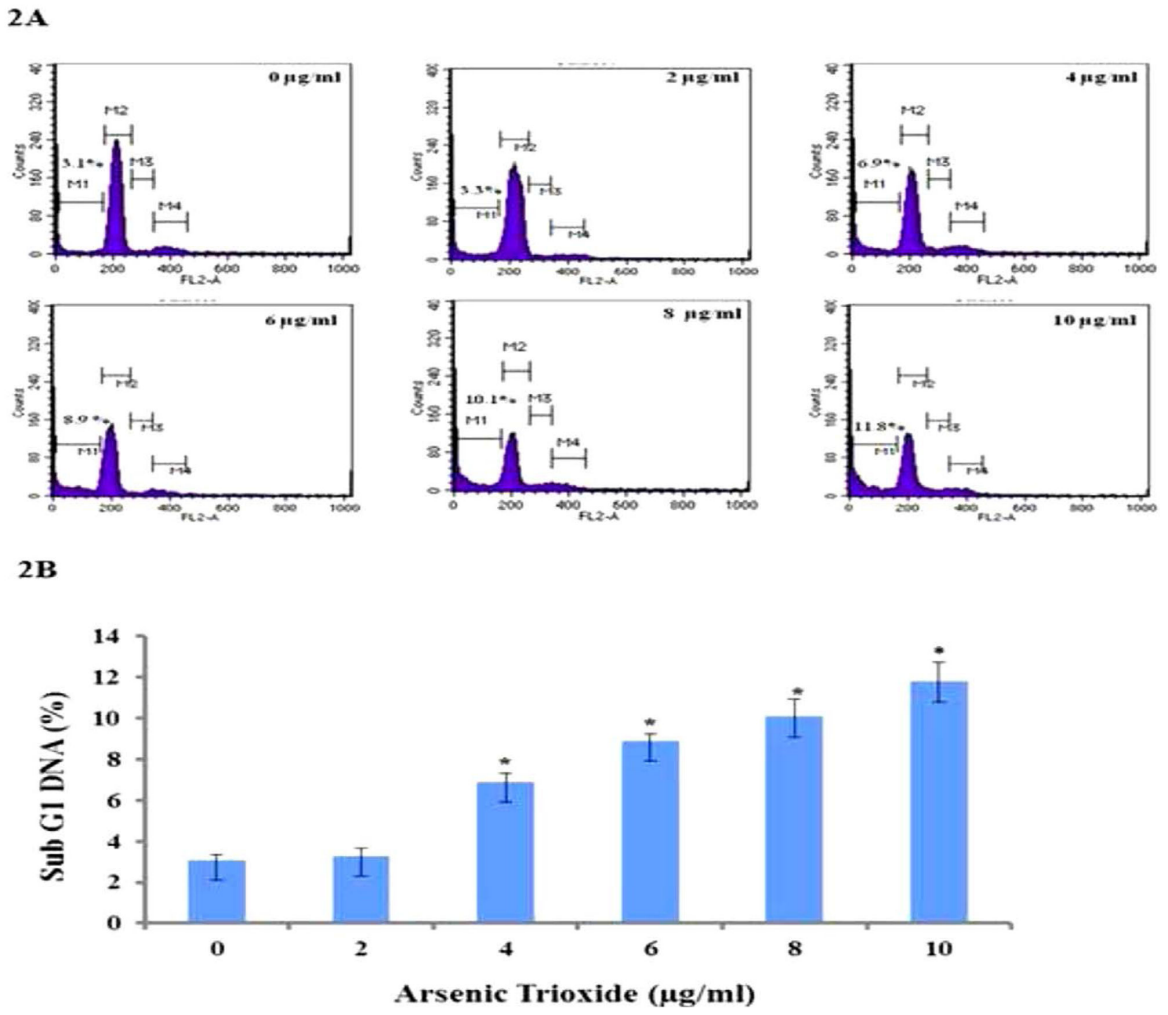
Flow cytometry analysis of cell distribution (sub G1) of HT-29 cells. 2A) Representative histograms of cell cycle distribution depicting apoptosis of ATO-treated (0 μg/mL to 10 μg/mL) colon cancer cells exposed for 24 h. The apoptotic fraction, the sub G1 phase of the cell cycle, is represented on the histograms. 2B) The percentages of cells undergoing apoptosis represented by sub G1 (M1) were 3.1% ± 0.28%, 3.3% ± 0.36%, 6.9% ± 0.42%, 8.9% ± 0.33%, 10.1% ± 0.84%, and 11.8% ± 0.95% for 0, 2, 4, 6, 8, and 10 μg/mL respectively. The rate of apoptosis increased in a concentration-dependent manner and was statistically significant (*) at 4, 6, 8 and 10 μg/mL relative to the controls.

Cell cycle analysis was conducted by flow cytometry to determine the effect of ATO on cell cycle progression by measuring the DNA content of cells at the sub G1 phase which represents cells undergoing apoptosis. The histograms in Figure 2A show the cell cycle distribution of HT-29 cells treated with ATO at 0, 2, 4, 6, 8 and 10 μg/ml for 24 h. The apoptotic fraction (sub G1 phase of the cell cycle) was represented by the M1 peak on the histograms. The other phases of the cell cycle (Go/G1, S and M) were represented on the histogram as M2, M3 and M4, respectively. DNA flow cytometric analysis indicated that ATO treatment of 0 and 2 μg/ml did not significantly (p>0.05) alter the cell cycle distribution. The percentages of cells undergoing apoptosis, represented by sub G1 (M1), were 3.1% ± 0.28%, 3.3% ± 0.36%, 6.9% ± 0.42%, 8.9% ± 0.33%, 10.1% ± 0.84%, and 11.8% ± 0.95% for 0, 2, 4, 6, 8, and 10 μg/mL respectively (Figure 2B). These results indicated that there were significant differences in the sub G1 phase in ATO-treated (4 μg/ml to 10 μg/ml) cells compared to the control (0 μg/ml). A concentration-dependent effect of ATO in the induction of sub G1 phase was observed and clearly demonstrated that ATO induced apoptosis in HT-29 cells.

### Assessing ATO-induced apoptosis using Annexin V-FITC/Propidium iodide

**Figure 3:**
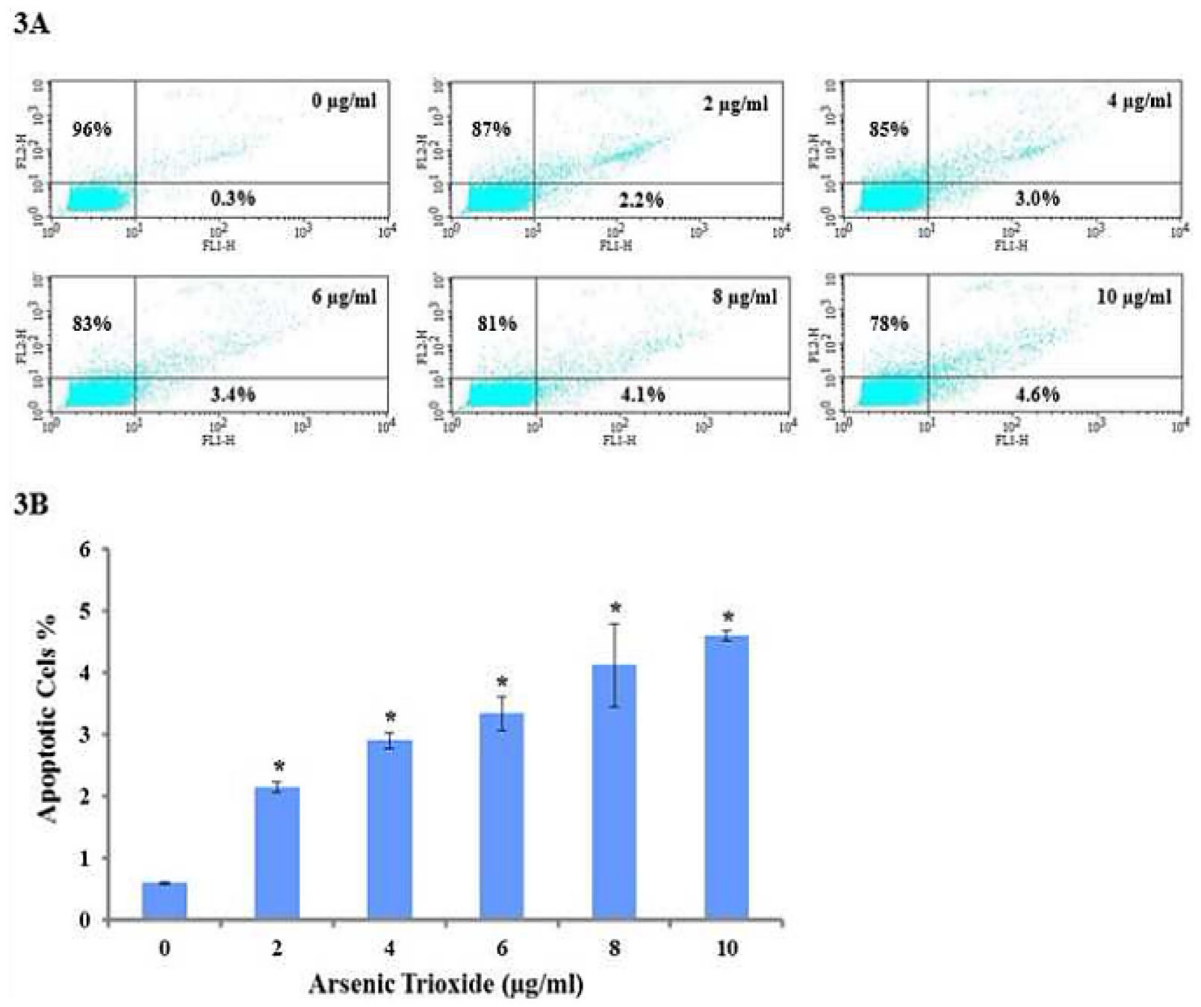
Evaluation of apoptosis using flow cytometry. 3A) Representative data from the Annexin V FITC flow cytometry analysis of ATO-treated HT-29 cells revealed that the apoptotic cells were significantly different (p<0.05) compared to the control. 3B) ATO induced apoptosis in HT-29 cells. Annexin V conjugated with PI revealed apoptotic cells as represented in lower right quadrants.

The induction of apoptosis in HT-29 cells exposed to ATO was also quantified using the Annexin V-FITC/PI assay. Flow cytometry analysis of HT-29 cells stained with Annexin VFITC conjugates and PI showed a significant increase (p<0.05) in apoptotic cells with increasing concentrations of ATO as shown in Figure 3. With reference to the untreated control, the number of viable cells decreased in population (Figure 3A) in a concentration-dependent manner. The proportion of living cells (Annexin V-FITC and PI-negative) was 96%, 87%, 85%, 83%, 81%, and 78% in ATO-treated cells 0, 2, 4, 6, 8, and 10 μg/ml. The proportion of early apoptotic cells (Annexin V-FITC-positive and PI-negative) was 0.3%, 2.2%, 3.0%, 3.4%, 4.1%, and 4.6% in the ATO-treated cells (0 μg/ml to 10 μg/ml). These results suggest that ATO induces early apoptosis in HT-29 cells in a concentration-dependent manner (Figure 3B).

### Induction of morphological changes of HT-29 cells

**Figure 4:**
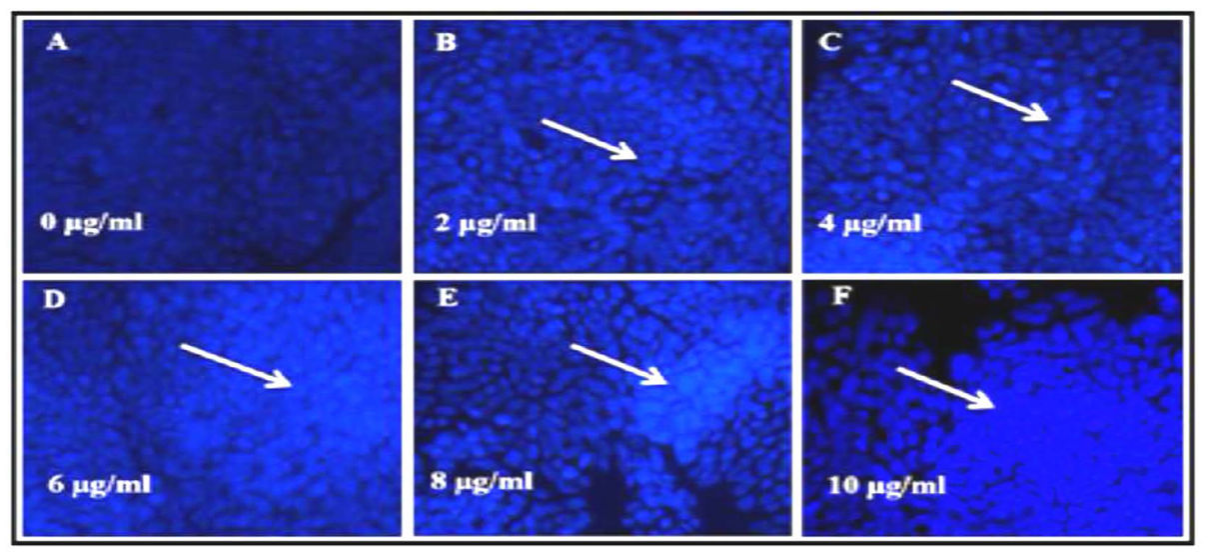
DAPI staining of HT-29 cells. HT-29 cells were treated with ATO (0 μg/ml to 10 μg/ml) for 24 h and stained by DAPI. Almost all cells in the control group (0 μg/ml) were normal. However, apoptotic cells appeared in a concentration-dependent manner after 24 h treatment with ATO (Magnification, 20X).

Untreated HT-29 cells grew well as observed by phase contrast and fluorescence microscopy. After 24 h of treatment, ATO produced apoptosis in a dose-dependent manner, and caused the cells to detach from the culture plates/substrates. ATO-treated HT-29 cells stained with DAPI showed condensed and fragmented nuclei, which are typical morphological features of apoptotic cells (Figure 4).

### Role of specific cellular proteins in ATO-induced apoptosis in HT-29 cells

**Figure 5:**
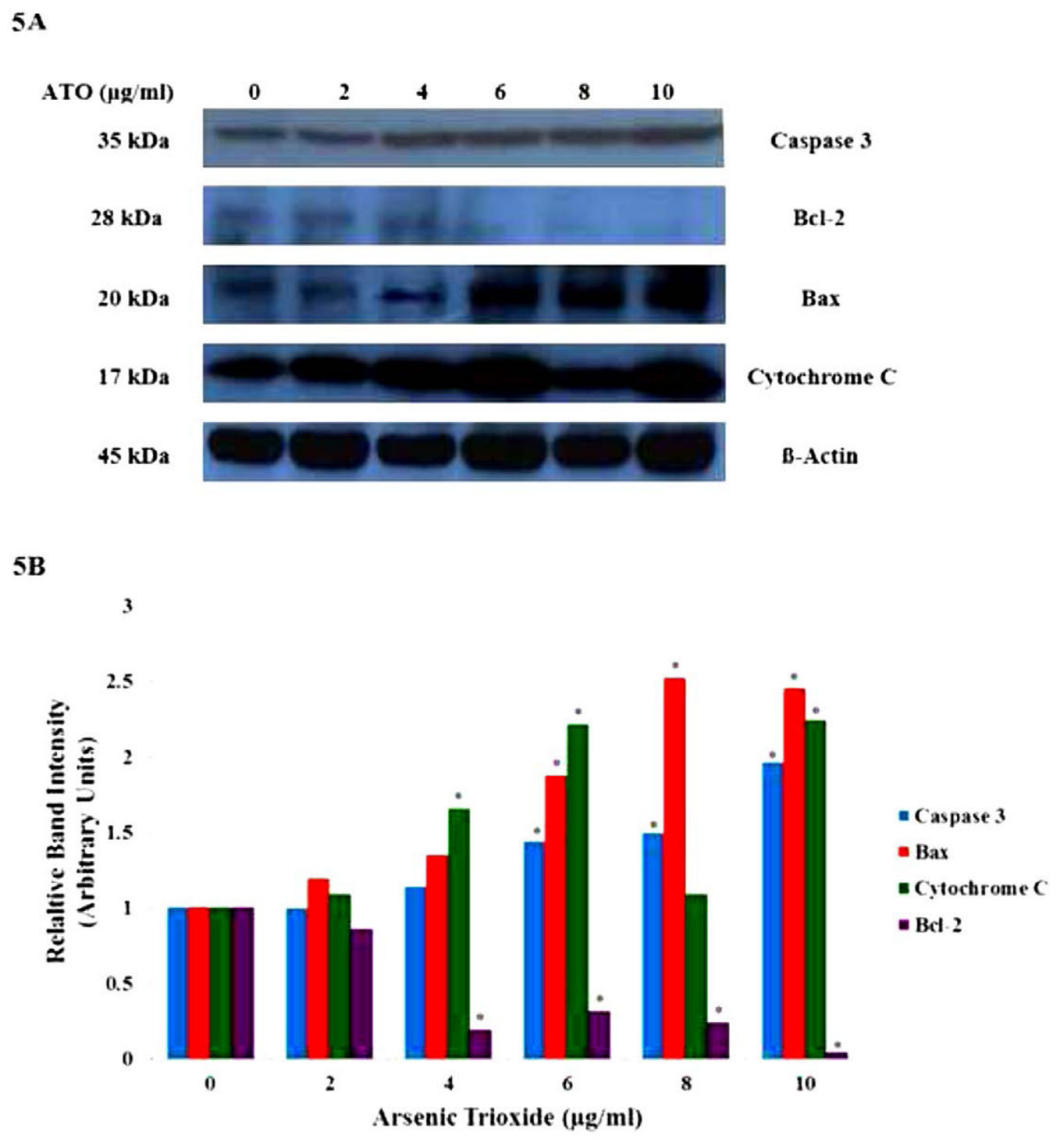
Effects of ATO on apoptosis-related proteins. HT-29 cells were treated with ATO (0 μg/ml to 10 μg/ml) for 24 h, and the expression levels of caspase 3, Bax, cytochrome C and Bcl-2 were determined by western blotting and densitometric analysis. B-actin was used as the internal control. (A) Western blots of intrinsic apoptotic pathway proteins in control and ATO-treated HT-29 cells for 24 h. ATO exposure significantly increased the expression levels caspase 3, Bax, and cytochrome C and decreased the expression level of Bcl-2 in a concentration-dependent manner. (B) Densitometric analysis of ATO-induced apoptotic protein expressions in HT-29 cells. Data represent the means of the three independent experiments ± SDs (*significantly different at p<0.05).

To ascertain the underlying mechanisms responsible for apoptosis, we examined the effects of ATO on the expression of apoptosis-related genes and proteins. The protein expression levels of Bcl-2 family proteins, including anti-apoptotic members such as Bcl-2, and pro-apoptotic members such as Bax, cytochrome c and caspase 3, were assessed by western blot analysis. Significant changes in the protein levels were observed in human colon carcinoma cells treated with various concentrations of ATO for 24 h. As shown in Figure 5A, there was an increase in the expression of both Bax and cytochrome c, and a decrease in the expression of Bcl-2 in ATO treated HT-29 cells compared to control cells. This demonstrates that ATO activates the mitochondrial apoptotic pathway in HT-29 cells via regulation of the expression of both pro- and anti-apoptotic proteins. The caspase cascade is one of the most important events in the process of apoptosis through the mitochondrial pathway. The study results also demonstrated that ATO upregulated the expression of caspase 3 (Figure 5A). Densitometric analysis showed a significant upregulation (p<0.05) of cytochrome C at 4, 6 and 10 μg/ml, while caspase 3 and Bax at 6 μg/ml to 10 μg/ml ATO (Figure 5B). In contrary, a significant down-regulation (p<0.05) of Bcl-2 expression was observed at 4 μg/ml to 10 μg/ml ATO treatment (Figure 5B). These results suggest the involvement of the Bax/Bcl-2-mediated caspase-3 pathway as well as the mitochondria or intrinsic pathway in ATO-induced apoptosis in HT-29 cells.

## Discussion

Oxidative stress, defined as a disturbance in the balance between the production of reactive oxygen species (free radicals) and antioxidant defenses. Reactive oxygen species (ROS) plays a vital role in various cellular biological activities including proliferation, growth, apoptosis, an invasion [48]. Published studies have shown that arsenic induces the generation of reactive oxygen species [49,50]. ROS can react with the polyunsaturated fatty acids of lipid membranes and induce lipid peroxidation. Heavy metals including arsenic and oxygen radicals play an important role in the peroxidation process. The process of lipid peroxidation is initiated by the attack of a free radical produced by a heavy metal such as arsenic, on unsaturated lipids and the resulting chain reaction is terminated by the production of lipid breakdown products, lipid, alcohols, aldehydes and malondialdehyde [51]. Oxidative stress was assessed by the lipid peroxidation assay measuring the formation of malondialdehyde (MDA), an end product from the peroxidation of polyunsaturated fatty acids in the plasma membrane [48] and can indicate oxidative damage in cells and tissues. The present study showed an increased level of MDA concentration in ATO-treated HT-29 cells, a finding consistent with studies with A549 and HL-60 cells treated with ATO [49,50], indicating that oxidative stress plays an important role in arsenic induced toxicity and cell damage.

Oxidative stress activates numerous major signaling pathways. Being highly reactive by nature, ROS can directly or indirectly modulate the functions of many enzymes through a multitude of signaling cascades which can influence cell survival or death. Arsenic trioxide has been known to induce oxidative stress which leads to the development of apoptosis [51,52]. It has been reported that severe oxidative stress ultimately causes cell death via either apoptotic or necrotic mechanisms [53]. Several studies [54,55] have reported similar increase in lipid peroxidation products (MDA) when treated with heavy metals.

Apoptosis, a phenomenon of programmed cell death, is a self-destruction mechanism involved in a variety of biological events. The mitochondria plays a crucial role in regulating cell death pathways [56]. Reactive oxygen species are mainly produced in the mitochondria and pay an important role in apoptosis. Apoptosis is one of the body’s major defense mechanism against cancer and is an active gene-directed tool for understanding developmental biology and tissue homeostasis [57–60]. Several morphological and biochemical features have been identified that characterizes an apoptotic cell. Morphologically, apoptosis is characterized by cell shrinkage, apoptotic bodies, chromatin condensation and membrane blebbing. Arsenic trioxide is an effective therapeutic tool in the treatment of APL and some solid tumors [15–18]. Arsenic trioxide has been reported to affect many biological processes such as cell proliferation, apoptosis, cell cycle progression, differentiation and angiogenesis [12,13]. Numerous studies have reported that ATO induces various effects in tumor cell lines [22–26], but the further detailed mechanisms underlying the different effects ATO are still elusive. In this study, we studied the influence of ATO on cell cycle distribution and apoptosis in human colon carcinoma HT-29 cells.

Arsenic trioxide has been shown to act on cells by influencing cell cycle arrest [61,62]. In the current study, we investigated the apoptotic effects of ATO in vitro on the human colon cancer cells by cell cycle distribution and apoptosis by flow cytometry. The significant difference in the sub G1 phase cell population between the control and ATO-treated HT-29 was observed. The sub G1 phase of the cell cycle is an important period where various signals interact to determine the proliferation, quiescence, differentiation, or apoptosis of cells [63,64]. In the present study, we demonstrated that a dose-dependent effect of ATO in the induction of sub G1 phase was observed and clearly demonstrated that ATO induced apoptosis in HT-29 cells. Arsenic trioxide has been shown to inhibit the proliferation of human lung cancer (A549) cells via cell cycle arrest and the induction of apoptosis [65]. Although ATO has been an effective treatment for the acute promyelocytic leukemia, the mechanism by which ATO induces cell death remains poorly understood. Studies have shown that ATO treatment resulted in cell-cycle arrest in human cancer cell lines such as MCF-7 [66] and As4.1 juxtaglomerular cells [67]. Arsenic trioxide blocked the cell cycle in G1 or at G2/M depending on the cell line.

Annexin V-FITC/Propidium assay provides a simple and effective method to detect one of the earliest events in apoptosis, the externalization of phosphatidylserine (PS), in living cells. In the early stages of apoptosis, the membrane phosphatidylserine (PS) is translocated from the inner to the outer plasma membrane, thereby exposing PS to the binding of annexin V [60,68–70]. The apoptotic markers of annexin V binding and apoptotic sub G0/G1 were dramatically increased in ATO treated HL-60 cells [66]. Although the mechanism by which ATO exerts its toxic effect in this cell line have not been fully elucidated, previous studies in our laboratory have demonstrated a dose-dependent response with regard to ATO toxicity to HT-29 cells. To determine whether ATO-induced toxicity in HT-29 cells is mediated by apoptosis and/or necrosis, we measured the Annexin V FITC/PI staining using the flow cytometry analysis. Annexin V FITC/PI assay helps to distinguish between apoptotic and necrotic cell death by identifying apoptosis at an earlier stage based on phosphatidylserine externalization prior to the measurement of nuclear changes such as DNA strand breaks. Annexin V binds to the membrane phospholipid phosphatidylserine that is located within the plasma membrane of apoptotic cells and PI stains the cellular DNA of those that have a compromised cell membrane [68–70]. Moreover, the cell population can be further categorized into early apoptotic cells, secondary necrotic cells (late apoptotic cells) as well as necrotic cells through the concurrent usage of PI as an exclusion dye. Data generated from this study revealed that ATO induces apoptosis in HT-29 cells in a dose-dependent manner. The percentage of cells stained with Annexin V (positive cells) and PI (necrotic cells) significantly (p<0.05) increased with the increasing concentrations of ATO (Figure 3). Consistent with findings in our laboratory by Walker et al. [25] which demonstrated that ATO induced apoptotic cell death in MCF-7 cells by annexin V-FITC staining. In addition, studies have shown that ATO induces cellular apoptosis in HL-60 promyelocytic leukemia cells in a dose-dependent manner, showing an increased expression of annexin positive cells in ATO treated cells compared to the control [70].

Caspases are known to be important mediators of apoptosis in both the intrinsic and extrinsic pathways. Bcl-2 (anti-apoptotic) and Bax (pro-apoptotic) are members of the Bcl-2 family of genes and are involved in the activation of caspases [34]. Futhermore, caspases are a family of proteins that are central effectors of apoptosis and their activation is also a hallmark of apoptosis. As a member of the caspases family, caspase-3 is known as the crucial executioner caspase by processing of its substrates that lead to morphological changes associated with apoptosis including DNA degradation, chromatin condensation, and membrane blebbing. In the current study, we believe that the ATO induced apoptosis through the mitochondrial pathway acts through Bcl-2. In this pathway, the outer mitochondrial membrane becomes permeable in response to apoptotic stimuli which releases cytochrome C. Cytochrome c binds to and activates procaspase-3, which is cleaved into caspase-3. Bcl-2 is an upstream effector molecule in the apoptotic pathway and a potent suppressor of apoptosis. It can oligomerize Bax, which subsequently depolarizes the mitochondrial membrane potential to release cytochrome C and induce apoptosis [34]. Previous studies showed that ATO activated Bax and cytochrome C expression and down-regulated Bcl-2 protein expression in HL-60 cells in a dose-dependent manner [70]. Previous studies reported that ATO-induced apoptosis was associated with the down-regulation of Bcl-2 protein in NB4 cells and the activation of Bax protein expression in lymphoma B-cells. In this study, we investigated the anti-cancer mechanism of ATO in human colon carcinoma cells. Our findings showed that ATO reduced the expression of Bcl-2 and increased the expression of Bax in HT-29 cells in a dose-dependent manner. In contrast, ATO-induced apoptosis was associated with activation of caspase 3 and cytochrome C in HT-29 cells in a dose-dependent manner.

## Conclusions

In summary, our results indicated that ATO-induced apoptosis in HT-29 cells is associated with malondialdehyde formation, phosphatidylserine externalization, capase-3 activation and the down-regulation of Bcl-2. Although the mechanism of action of ATO is not well understood, our study has shown that oxidative stress, a biomarker of cellular injury, may be a potential mechanism for apoptosis of ATO and demonstrates a pivotal role of the mitochondria in cell death of HT-29 cells. Taken together our findings indicate that ATO has a substantial bioactivity against HT-29 cells. However, further investigations using animal models of colon cancer are needed to provide new insights into the potential application of ATO in the treatment of colon cancer.

## Abbreviations

ANOVA: One Way Analysis of Variance
APL: Acute Promyelocytic Leukemia
ATCC: American Type Culture Collection
ATO: Arsenic Trioxide
BCA: Bicinchoninic Acid
DAPI: 4′,6-Diamidino Phenylindole
ECL: Enhanced Chemiluminescence
FACS: Fluorescence Activated Cell Sorting System
FBS: Fetal Bovine Serum
FITC: Fluorescein Isothiocyanate
HRP: Horseradish Peroxidase
MDA: Malondialdehyde
MTT: 3-(4,5-Dimethyl-2-Thiazolyl)-2,5-Diphenyl-2,Tetrazoliumbromide
OS: Oxidative Stress
PI: Propidium Iodine
ROS: Reactive Oxygen Species
SDS-PAGE: Sodium Dodecyl Sulfate Polyacrylamide Gel Electrophoresis
PS: Phosphatidylserine
PVDF: Polyvinylidene Difluoride
SD: Standard Deviation
UV/Vis: Ultraviolet Visible Spectroscopy
